# Kenya’s Healthcare Crisis: Consequences of Delayed Deployment of Medical and Dental Graduates to Internship Centres

**DOI:** 10.34172/ijhpm.9034

**Published:** 2025-08-17

**Authors:** Kosasia O’Neil Wamukota

**Affiliations:** AIC Kijabe Hospital, Kijabe, Kenya.

## Introduction

 In 2016, the World Health Organization (WHO) estimated that 18 million more health workers would be needed, particularly in low- and middle-income countries, to achieve universal health coverage (UHC) by 2030.^1^ To achieve this, the WHO recommended, among other strategies, the strengthening of career pathways for health workers. Globally, an internship is the entry point for healthcare professionals’ career pathways.^2^

 However, internship programs for healthcare professionals in low- and middle-income countries are increasingly constrained by financial, administrative, and policy barriers. For instance, in India, foreign-trained medical graduates have faced up to 10-month delays in internship placement.^3^ In South Africa, funding shortages in government hospitals have delayed internship placement for junior doctors.^4^ In Ghana, pharmacy house officers have worked for nearly a year without allowances.^5^ In Nigeria, medical and pharmacy graduates have reported having to pay bribes to secure internship placements.^6^ In Uganda, a shortage of government funding has led to indefinite delays in deploying medical interns.^7^ Collectively, these examples highlight how weak internship systems hinder the development of health workers and weaken national health systems, thus undermining progress toward UHC.

 The internship program for junior doctors in Kenya faces similar systemic challenges. This article focuses on the delayed deployment of medical and dental graduates to internship centres in Kenya, examining its root causes, impact on trainees and the healthcare system, and potential solutions.

## Internship for Medical and Dental Graduates in Kenya

 An internship is a mandatory, year-long period of supervised employment for medical and dental graduates in Kenya. During this period, they consolidate their knowledge, skills, and attitudes to fulfil registration requirements and be competent practitioners.^8^ Medical interns rotate through internal medicine, surgery, paediatrics and child health, obstetrics and gynaecology, and mental health departments.^8^ In contrast, dental interns must demonstrate competence in the following disciplines by the end of their internship: oral and maxillofacial surgery, conservative and prosthetic dentistry, orthodontics and pediatric dentistry, and periodontology.^8^ All medical and dental interns must participate in community health practice. Upon completing their internship, medical and dental graduates apply for registration and licensure through the Kenya Medical Practitioners and Dentists Council (KMPDC). KMPDC also coordinates the oath-taking and placement of medical and dental graduates into various internship centres.^8^ The Ministry of Health (MoH) is responsible for deploying graduates to these centres.

 Despite its importance, medical and dental internships in Kenya are plagued by systemic challenges, including delayed deployment to internship centers, excessive workload, inadequate resources in hospitals, inadequate supervision, and unemployment post-internship. Many interns, especially in public hospitals, report excessive workload due to staff shortages.^9^ They do not have adequate resources to support safe and effective patient care. Some also face poor supervision due to limited availability of consultants, thus undermining learning and professional growth.^9^ Beyond internship, transition into permanent employment is not guaranteed due to structural inefficiencies in Kenya’s devolved healthcare system. Systemic barriers such as delayed hiring, opaque recruitment processes, and political interference at the county level prevent the absorption of junior doctors after internship.^10^

 Among these challenges, delayed deployment to internship centers has emerged as an especially pressing concern in recent years. Even though policy dictates that medical and dental graduates in Kenya should be deployed to internship centres within one month of completing their undergraduate training, regardless of graduation dates, this timeline has not been consistently upheld.^8^ The 2023 cohort of graduates had the most prolonged delay, with most waiting nearly a year before deployment.^11^

## Causes of Delayed Internship Deployment

 Delays in deployment arise from a combination of factors, including a growing pool of graduates, budgetary shortfalls, collective bargaining disputes, and institutional bottlenecks.

 The number of medical and dental graduates from public and private universities in Kenya has been increasing steadily over the years. The number of medical graduates almost doubled between 2016 and 2019, from 320 to 628 graduates, while the number of dental graduates nearly tripled during the same period, from 25 to 69 graduates.^12^ This rapid increase in the graduate pool has not been matched by a proportional increase in government funding for internship deployment. In the 2024/2025 fiscal year, the MoH requested 4.8 billion Kenya shillings to deploy 3760 health professional interns, among them 849 medical and 72 dental graduates.^13^ However, it received only 3.7 billion Kenya shillings, leaving a funding shortfall of 1.1 billion Kenya shillings.^13^ This shortfall directly contributed to delays in deploying interns.

 In early 2024, the Cabinet Secretary for Health proposed slashing health professional intern salaries based on an advisory by the Salaries and Remuneration Commission to reduce government spending. Both medical and dental interns were to receive 70 000 Kenya shillings monthly from 206 400 Kenya shillings.^13^ The proposed salary cuts contravened the Collective Bargaining Agreement signed in 2017, leading to a 56-day national strike by doctors, pharmacists, and dentists, thus delaying internship deployment.^13^ The matter was also contested in court, further delaying internship deployment.

 Institutional bottlenecks also play a role in delayed internship deployment. The key institutions involved in deploying medical and dental graduates to internship centers are KMPDC and the MoH.^8^ Medical and dental graduates are placed into internship centers randomly through an online system. While this digital system improves transparency and efficiency, some graduates are often dissatisfied with the internship centres in which they are placed. Therefore, there is usually a window for them to appeal for a change in placement. This appeals phase must be completed before the final validated list of interns and their internship centres is submitted to the MoH for deployment. This multistep process introduces delays in deployment, especially when there is a high volume of appeals.

## Consequences of Delayed Internship Deployment

###  Impact on Medical and Dental Graduates

 Delayed deployment of medical and dental graduates to internship centres hinders career progression, leads to decay of knowledge and skills, and creates emotional distress among graduates.

 An internship is mandatory for medical and dental graduates in Kenya to be licensed to practice independently.^11^ Delayed deployment delays licensure, thus hindering graduates from advancing their careers. They can neither apply for permanent medical or dental positions nor pursue specialization. Postgraduate training programs often require candidates to have completed their internship, and sometimes have post-internship work experience. The monthly salary, medical and dental interns receive during internship supports their professional development by allowing them to attend conferences, seminars, and workshops, which are often costly.^14^ Delays in deployment, therefore, deprive graduates of critical financial resources needed for career growth.

 The main internship objective for medical and dental graduates in Kenya is to consolidate their knowledge, skills, and attitudes to be competent practitioners.^8^ Their knowledge and clinical skills decline when they are not deployed promptly. After years of rigorous academic training, graduates risk forgetting critical information without regular application. They also experience decay of clinical skills due to the lack of application of these skills and evolving scientific evidence.^15^

 Delayed deployment also adversely affects the mental well-being of medical and dental graduates. After graduating from medical or dental school, one expects to transition smoothly into an internship. Indefinite delays in deployment create anxiety and uncertainty among the graduates about their careers.^16^ They may experience feelings of helplessness and despair as they watch their peers progress in their careers while they stagnate.

###  Broader Implications for Kenya’s Healthcare System

 At a systemic level, delayed deployment of medical and dental graduates to internship centres worsens the critical shortage of healthcare workers, and promotes brain drain, thus reducing the quality of care patients receive.

 As of 2015, the doctor-to-patient ratio in Kenya was 1:5263, 5 times higher than the WHO recommended ratio of 1:1000.^17^ Medical and dental interns are critical in bridging this gap, serving as essential frontline healthcare providers in public hospitals. They are often the first healthcare workers to interact with patients in public hospitals and perform the initial evaluation, diagnosis, and treatment.^8^ Delayed deployment of medical and dental interns worsens the strained doctor-to-patient ratio, increasing the workload for an overstretched healthcare workforce. This can lead to burnout, longer patient waiting times, and delayed diagnosis, compromising patient care.^18^

 Delayed deployment to internship centres also promotes brain drain. One of the reasons for health professionals’ brain drain in Kenya is a lack of career development opportunities.^19^ An internship is the entry point to the career of medical doctors and dentists in Kenya. Graduates may forfeit an internship in Kenya and seek opportunities abroad where internship placements are faster and more predictable. This emigration worsens the already low doctor-to-patient ratio in Kenya and places additional stress on the remaining workforce.^19^

## Strategies to Prevent Delayed Internship Deployment

 With the number of medical and dental graduates in Kenya increasing annually, delays in internship deployment are likely to become even more prolonged if left unaddressed. Strategies that should be considered to address this issue include proper budgeting through forecasting graduate numbers, expanding internship sites, especially in the private sector, establishing a centralized internship management system, and strengthening legal frameworks on deployment timelines.

 The KMPDC indexes new medical and dental students to track their progress throughout pre-service education until graduation.^17^ Medical and dental schools regularly update KMPDC on student progress and expected completion dates. The MoH should use this data to project the number of graduates each year and budget for their timely deployment before they graduate to minimize last-minute financial constraints that have often been used as a reason for delayed deployment.^13^

 According to the KMPDC website, there are 76 approved internship centres for medical interns and 8 for dental interns.^20^ All the approved internship centres for dental interns and most approved internship centres for medical interns are public hospitals, while a few are private and faith-based hospitals. Overreliance on public hospitals increases the budget required by the MoH to deploy interns. The KMPDC should approve more internship centres, particularly private hospitals that meet the necessary standards. This will ease the financial burden on the MoH because private hospitals are responsible for the remuneration of their interns, thus preventing unnecessary delays in deployment.

 A centralized internship management system should be developed to address institutional bottlenecks in deploying interns. Countries such as Nigeria have such a system, which helps improve efficiency in deploying house officers to house jobs. This portal will integrate workflows between KMPDC and MoH. It will allow interns to track their progress from oathing to deployment. It will also enable them to appeal for a change in the internship placement within a specified timeframe and track the outcome of their appeals. The portal should allow data sharing between KMPDC and the MoH. This will minimize manual processes that delay the transfer of the validated list of interns and their placement centres to MoH for deployment.

 Even though KMPDC recommends that medical and dental graduates be deployed within one month of completing their undergraduate training, there is no legal mechanism to enforce this provision, making it ineffective.^8^ In the past, there were minimal delays in deploying interns; hence, there was no need for a legal mechanism to enforce the policy on deployment timelines. However, as the delays have become more rampant in the recent past, the Medical Practitioners and Dentists Act should be amended to explicitly require the MoH to deploy interns within one month of completing their undergraduate training. The Parliamentary Committee on Health should monitor how well the MoH adheres to this timeline and identify and address any obstacles causing delays.

## Conclusion

 Delayed deployment of medical and dental graduates in Kenya to internship centres is more than an administrative hiccup. It is a crisis that profoundly impacts the graduates and the healthcare system. For graduates, it leads to career stagnation, deterioration of knowledge and clinical skills, and mental health challenges. For the healthcare system, it exacerbates workforce shortages and promotes brain drain, thus compromising patient care. It is not a unique Kenyan problem, as other low- and middle-income countries face a similar situation. These countries must invest in well-coordinated health professional internship programs that ensure a timely transition from undergraduate education to practice. Strategic planning, adequate funding, stronger legal frameworks, and cross-sectoral collaboration are essential to safeguard young health professionals’ careers, thus building resilient health systems capable of achieving UHC.

## Ethical issues

 Not applicable.

## Conflicts of interest

 Author declares that he has no conflicts of interest.

## Disclaimers

 The views expressed in this article are solely those of the author and do not represent the views of any affiliated institutions or organizations.
